# CYLD dysregulation in pathogenesis of sporadic inclusion body myositis

**DOI:** 10.1038/s41598-019-48115-2

**Published:** 2019-08-12

**Authors:** Satoshi Yamashita, Yoshimasa Matsuo, Nozomu Tawara, Kentaro Hara, Masanori Yamamoto, Tomo Nishikami, Kensuke Kawakami, Xiao Zhang, Ziwei Zhang, Tsukasa Doki, Yukio Ando

**Affiliations:** Department of Neurology, Graduate School of Medical Sciences, Kumamoto University, 1-1-1 Honjo, Chuo-ku, Kumamoto 860-8556 Japan

**Keywords:** Neuromuscular disease, Cellular neuroscience

## Abstract

Sporadic inclusion body myositis (sIBM) is the most commonly acquired myopathy in middle-aged and elderly people. The muscle histology is characterized by both inflammation and degeneration, including sarcoplasmic aggregation of TDP-43. Cylindromatosis (CYLD) is a deubiquitinating enzyme that targets Lys63-linked ubiquitin chains and negatively regulates signal transduction pathways, such as NF-κB signalling pathways. We examined localization of CYLD as well as phosphorylated TDP-43, phosphorylated p62, and Lys63-linked ubiquitin in muscle tissues of sIBM patients and muscle-specific wild-type TDP-43 transgenic (TDP-43 TG) mice. We investigated whether overexpression of CYLD can affect muscle toxicity in the cell models treated by endoplasmic reticulum (ER) stress inducers tunicamycin and thapsigargin. CYLD expressed with phosphorylated TDP-43, phosphorylated p62, and Lys63-linked ubiquitin in the nuclear and perinuclear regions of muscle fibres of wild-type TDP-43 TG mice and the degenerative myofibres of sIBM patients with rimmed vacuoles and endomysial cellular infiltration. Although expression levels of CYLD decreased and cell viability was reduced in cells treated with ER stress inducers, wild-type CYLD, but not the catalytic mutant, substantially improved cell viability based on the deubiquitinase activity. Dysregulation of CYLD may reinforce myodegeneration in the pathophysiology of sIBM by attenuating autophagic clearance of protein aggregates. Regulating CYLD in muscle fibres might serve as a novel therapeutic strategy for sIBM treatment.

## Introduction

Sporadic inclusion body myositis (sIBM) is the most commonly acquired myopathy in middle-aged and elderly people in Western countries and possibly also in Japan^1^. sIBM patients exhibit slowly progressive muscle weakness and degeneration of the quadriceps, and/or the finger and wrist flexor muscles. Immunosuppressive treatments have been disappointing, and many patients become wheelchair-bound 7–10 years after onset of the disease^2–4^.

The muscle histology of sIBM is characterized by inflammatory features such as mononuclear cells infiltrating the endomysium and non-necrotic myofibres, which has resulted in its classification as an autoimmune inflammatory disease like dermatomyositis and polymyositis; and degenerative features including formation of rimmed vacuoles and abnormal sarcoplasmic aggregation of proteins^5,6^. Recent histological investigations have shown that p62/SQSTM1 and TDP-43 accumulate frequently within damaged myofibres of sIBM tissues^7–9^. Nakano *et al*. showed that p62, lysine (Lys) 63-linked ubiquitin, and LC3 were collaboratively involved in selective autophagy against ubiquitinated protein aggregates in sIBM-affected tissues^10^. During this process, compromised binding of the p62-ubiquitinated protein complex to LC3 terminates the autophagic process at the initial stage, and then causes the formation of p62-oligomer aggregates with Lys63-ubiquitinated proteins.

Endoplasmic reticulum (ER) stress and the unfolded protein response (UPR) have been identified in the muscle fibres of sIBM tissues^11,12^. Activation of nuclear factor κB (NF-κB) has been shown to be an inflammatory response to the onset of sIBM^13,14^. Importantly, ER stress, induced by tunicamycin or thapsigargin treatment, resulted in NF-κB activation followed by induction of the myostatin precursor protein in a culture model of human muscle, mimicking the inflammatory and degenerative aspects of sIBM^14^.

Cylindromatosis (CYLD) was initially identified in the diagnosis of familial cylindromatosis, which is caused by mutations to the tumour suppressor *CYLD* gene^15^. CYLD is a deubiquitinating enzyme that targets Lys63-linked ubiquitin chains and negatively regulates signal transduction factors and pathways, including transforming growth factor-β (TGF-β) and NF-κB signalling pathways^16^. So far, CYLD has been found to regulate various biological pathways: including cell proliferation, survival, and migration; immune responses; osteoclastogenesis; and spermatogenesis^17^.

We previously reported that optineurin (OPTN) is involved in the pathophysiological mechanisms of skeletal muscular degeneration in cooperation with TDP-43 in sIBM^9^. OPTN has been shown to regulate NF-κB activation by mediating the interaction of CYLD with the ubiquitinated receptor interacting protein (RIP) in order to facilitate the deubiquitination of RIP^18^. Therefore, our study aimed to investigate whether dysregulation of CYLD is involved in sIBM by comparing muscle tissues of sIBM patients and muscle-specific wild-type TDP-43 transgenic (TDP-43 TG) mice, as well as myogenic cell lines treated with the ER stress inducers, tunicamycin and thapsigargin.

## Results

### Localization of CYLD in the muscle fibres of sIBM patients

**Figure 1:**
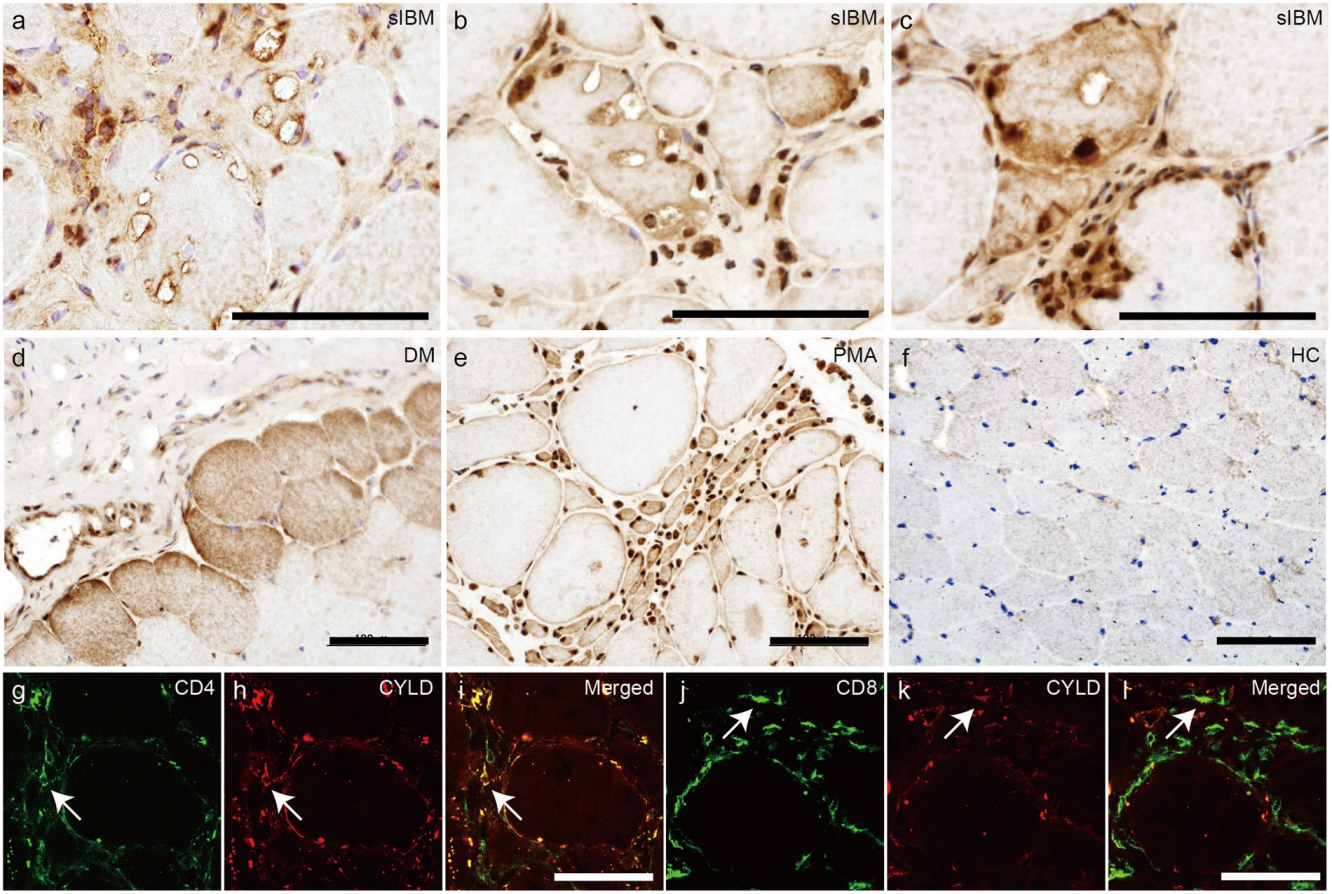
Localization of CYLD in muscle fibres of sIBM patients. (**a**–**f**) Representative immunohistochemical staining for CYLD using biopsy specimens of skeletal muscles from patients with sIBM (**a**–**c**), dermatomyositis (**d**), progressive muscular atrophy (**e**), and healthy control (**f**). Nuclei were stained with haematoxylin. Scale bars = 100 μm. (**g**–**l**) Confocal microscopic analysis of localisation of CD4 (**g**) or CD8 (**j**) and CYLD (**h**,**k**) in muscles of patients with sIBM. Merged images are presented in (**i**) and (**l**). Arrows indicate colocalization of CD4 or CD8 with CYLD. Scale bars = 50 μm.

We first compared the localization of CYLD in healthy control samples, control disease samples, and muscle samples from patients with sIBM. Our control disease samples consisted of patients with dermatomyositis, polymyositis, and progressive muscular atrophy. The immunohistochemical study revealed nuclear staining for CYLD in all samples (Table 1, Fig. 1). Ten of 11 sIBM patients showed CYLD localization in the cytoplasmic, perinuclear or surrounding regions of rimmed vacuoles in degenerative myofibres (Table 1, Fig. 1a–c), as well as invading and infiltrating cells in the endomysium (Fig. 1c). In contrast, CYLD was expressed diffusely in the sarcoplasm of atrophied myofibres in patients with dermatomyositis (Fig. 1d). CYLD expression was diffuse in the sarcoplasm of grouped atrophic muscle fibres from patients with progressive muscular atrophy (Fig. 1e). The healthy control showed faint sarcoplasmic staining for CYLD (Fig. 1f). Immunofluorescence analysis revealed colocalization of CD4 and CD8 signals with CYLD in endomysial cellular infiltration sites, suggesting that at least some subset of CD4- and CD8-positive T cells express CYLD (Fig. 1g–l).

**Table 1:** Clinical summary of the patients in the present study.

Case no.	Diagnosis	Age at biopsy (age at onset)	Sex	Max CK (U/l)	Biopsy	CYLD staining
1	sIBM	75 (50)	M	49	quadriceps	+(nuclear, cytoplasmic aggregates)
2	sIBM	60 (40)	F	348	biceps brachii	+(nuclear, perinuclear)
3	sIBM	70 (68)	M	497	quadriceps	+(nuclear, perinuclear)
4	sIBM	86 (74)	M	6686	quadriceps	+(nuclear)
5	sIBM	84 (79)	M	235	biceps brachii	+(nuclear, perinuclear, punctate)
6	sIBM	79 (77)	M	740	quadriceps	+(nuclear, perinuclear)
7	sIBM	72 (67)	M	539	biceps brachii	+(nuclear, perinuclear)
8	sIBM	67 (60)	M	1954	quadriceps	+(perinuclear)
9	sIBM	69 (55)	M	524	biceps brachii	+(punctate)
10	sIBM	72 (70)	M	339	biceps brachii	+(perinuclear)
11	sIBM	76 (74)	F	2692	deltoid	+(nuclear, perinuclear, punctate)
12	PM	58 (58)	F	4716	biceps brachii	+(nuclear, diffuse)
13	PM	54 (54)	F	3810	quadriceps	+(nuclear, diffuse)
14	PM	65 (65)	F	1273	biceps brachii	+(nuclear, diffuse)
15	DM	52 (51)	M	2521	deltoid	+(nuclear, diffuse)
16	DM	39 (39)	F	316	deltoid	+(nuclear, diffuse)
17	DM	27 (27)	F	4986	biceps brachii	+(nuclear, diffuse)
18	SBMA	55 (52)	M	1734	biceps brachii	+(nuclear, diffuse)
19	PMA	63 (58)	M	1029	biceps brachii	+(nuclear, diffuse)
20	ALS	83 (79)	F	384	gastrocnemius	+(nuclear, diffuse)
21	HC	54	F	—	biceps brachii	—
22	HC	58	F	—	biceps brachii	—
23	HC	45	F	—	biceps brachii	—

sIBM, sporadic inclusion body myositis; PM, polymyositis; DM, dermatomyositis; SBMA, spinobulbar muscular atrophy; PMA, progressive muscular atrophy; ALS, amyotrophic lateral sclerosis; HC, healthy control; CK, creatine kinase (normal range: 57–284 U/l).

### Accumulation of CYLD in the muscle fibres of TDP-43 TG mice

**Figure 2:**
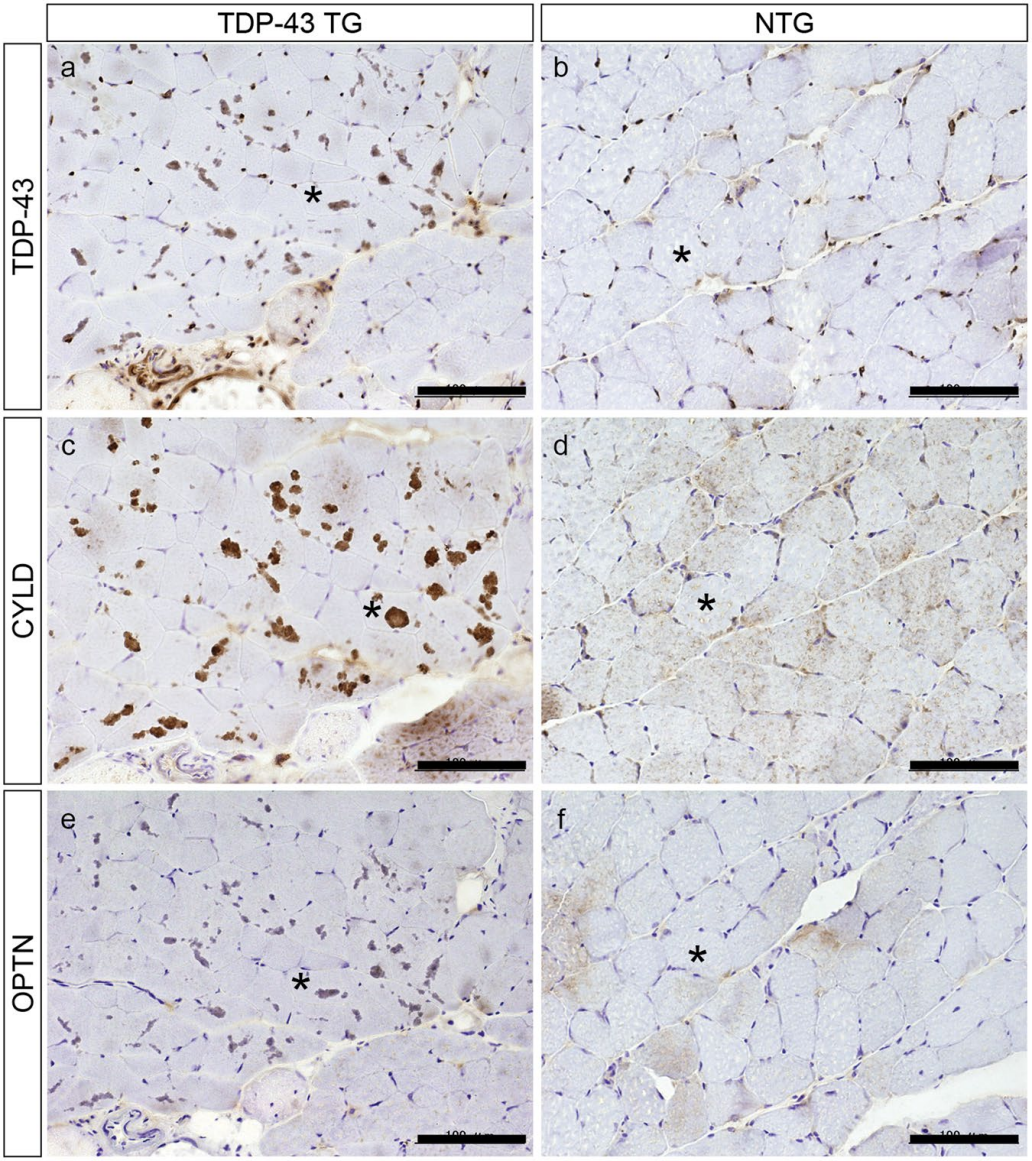
Aggregation of CYLD in muscle fibres of TDP-43 TG mice. Representative immunohistochemical staining for TDP-43 (**a**,**b**), CYLD (**c**,**d**), and optineurin (**e**,**f**) using muscle specimens from TDP-43 TG (**a**,**c**,**e**) and NTG mice (**b**,**d**,**f**). Nuclei were stained with haematoxylin. Asterisks indicate identical muscle fibres. Scale bars = 100 μm.

We next examined localization of CYLD in muscle tissues of TDP-43 TG mice. As shown in Fig. 2a, TDP-43 TG mice showed TDP-43-positive sarcoplasmic aggregation as well as nuclear staining, whereas non-transgenic littermates (NTG) only showed TDP-43 nuclear signals (Fig. 2b). Of note, immunohistochemistry for CYLD showed similar sarcoplasmic aggregates as observed in TDP-43 staining in myofibres of TDP-43 TG mice (Fig. 2c). However, some NTG mice myofibres showed a faint sarcoplasmic or nuclear pattern for CYLD. (Fig. 2d). Immunohistochemistry for OPTN also revealed similar sarcoplasmic aggregations in myofibres of TDP-43 TG mice (Fig. 2e) whereas NTG mice had only faint sarcoplasmic staining (Fig. 2f).

### Colocalization of CYLD with p-TDP-43, p-p62, and Lys63-linked ubiquitin in the muscle fibres of sIBM patients and TDP-43 TG mice

**Figure 3:**
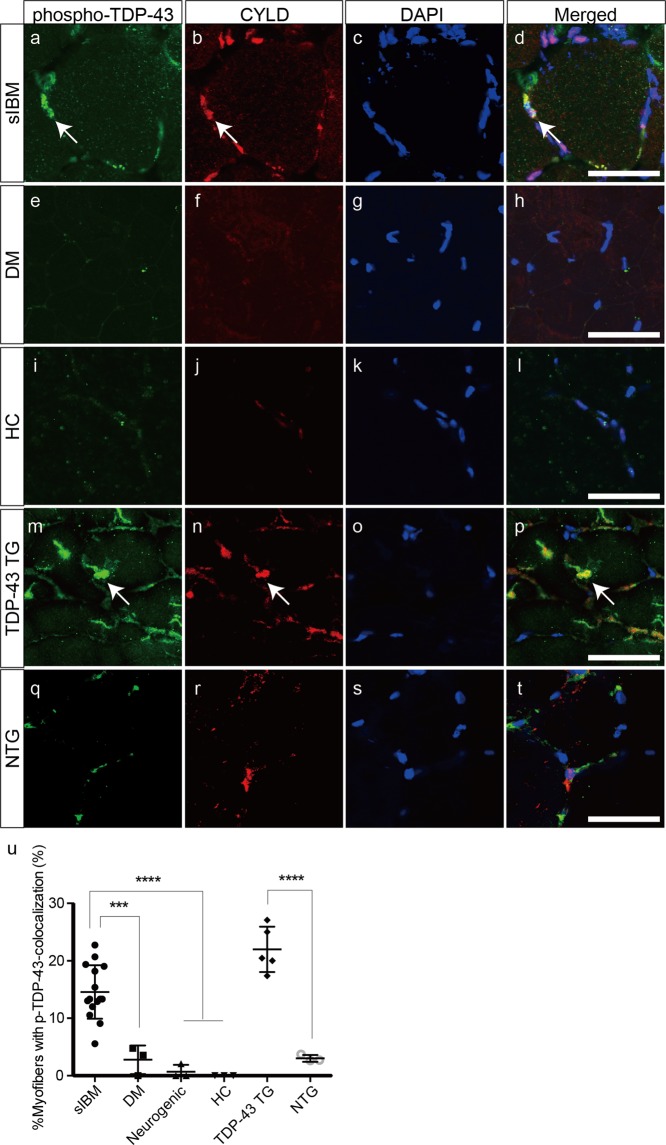
Colocalization of CYLD with phosphorylated TDP-43 in muscle fibres of sIBM patients and TDP-43 TG mice. (**a**–**t**) Confocal microscopic analysis of localization of phosphorylated TDP-43 (**a**,**e**,**i**,**m**,**q**) and CYLD (**b**,**f**,**j**,**n**,**r**) in muscles of patients with sIBM (**a**–**d**), dermatomyositis (**e**–**h**), healthy control (**i**–**l**), and TDP-43 TG (**m**–**p**) or NTG mice (**q**–**t**). Nuclei were stained with 4′,6-diamidino-2-phenylindole (DAPI). Merged images are presented in (**d**,**h**,**l**,**p**,**t**). Arrows indicate colocalization of phosphorylated TDP-43 with CYLD. Scale bars = 50 μm. (U) Percentages of myofibres with colocalization of phosphorylated TDP-43 and CYLD in each group. n = 3–15 per group. ***P < 0.001; ****P < 0.0001.

We then investigated localization between pathogenic TDP-43 and CYLD in muscle specimens from patients with sIBM and TDP-43 TG mice. Phosphorylated TDP-43 (p-TDP-43) colocalized with CYLD mainly in the nuclear and perinuclear areas (Fig. 3a–d). In contrast, p-TDP-43 expression was not detected in myofibres from patients with polymyositis, dermatomyositis (Fig. 3e), or the healthy control (Fig. 3i). Sarcoplasmic aggregates were stained with anti-p-TDP-43 antibodies in TDP-43 TG mice (Fig. 3m). These p-TDP-43-positive aggregates frequently colocalized with CYLD in muscle fibres (Fig. 3m–p). However, only faint signals for p-TDP-43 were observed in NTG mice (Fig. 3q), although CYLD localized in the nuclear area (Fig. 3r). Percentages of myofibres with colocalization of p-TDP-43 and CYLD were substantially higher in muscle samples of patients with sIBM and TDP-43 TG mice, compared to the other experimental groups (Fig. 3u).

**Figure 4:**
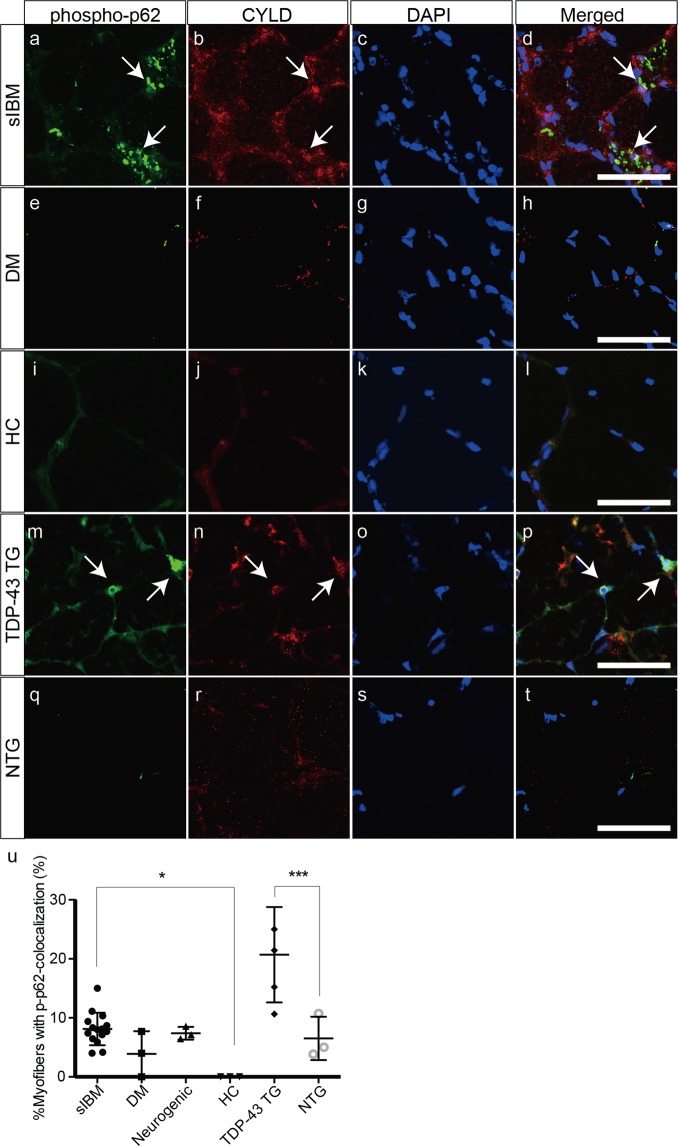
Colocalization of CYLD with phosphorylated p62 in the muscle fibres of sIBM patients and TDP-43 TG mice. (**a**–**t**) Confocal microscopic analysis of localization of phosphorylated p62 (**a**,**e**,**i**,**m**,**q**) and CYLD (**b**,**f**,**j**,**n**,**r**) in muscles of patients with sIBM (**a**–**d**) or dermatomyositis (**e**–**h**), healthy control (**i**–**l**), and TDP-43 TG (**m**–**p**) or NTG mice (**q**–**t**). Nuclei were stained with DAPI. Merged images are presented in (**d**,**h**,**l**,**p**,**t**). Scale bars = 50 μm. (**u**) Percentages of myofibres with colocalization of phosphorylated p62 and CYLD in each group. n = 3–15 per group. *P < 0.05; ***P < 0.001.

Phosphorylated p62 (p-p62) is a putative marker for selective autophagy in the pathogenesis of sIBM. Thus, we next examined whether p-p62 was associated with CYLD in muscle tissues of patients with sIBM and TDP-43 TG mice. P-p62 formed punctate aggregates in the sarcoplasm of sIBM tissues (Fig. 4a). Most of the p-p62-positive aggregates did not colocalize with CYLD in the sarcoplasm (Fig. 4a–d). Importantly, the nuclear and perinuclear p-p62-positive aggregates colocalized with CYLD (Fig. 4a–d). Fewer p-p62-positive aggregates were observed in muscles samples of patients with dermatomyositis (Fig. 4e), and the healthy control (Fig. 4I). P-p62 expression was observed in the nuclear and perinuclear areas of TDP-43 TG mice where CYLD colocalized with p-p62 (Fig. 4m–p), whereas NTG mice showed fewer aggregates of p-p62 (Fig. 4q). The percentage of myofibres with colocalization of p-p62 and CYLD was considerably higher in muscle samples of patients with sIBM than in the healthy control. Similarly, the percentage of myofibres with colocalization of p-p62 and CYLD was substantially increased in muscle samples of TDP-43 TG mice, compared to NTG mice (Fig. 4u).

**Figure 5:**
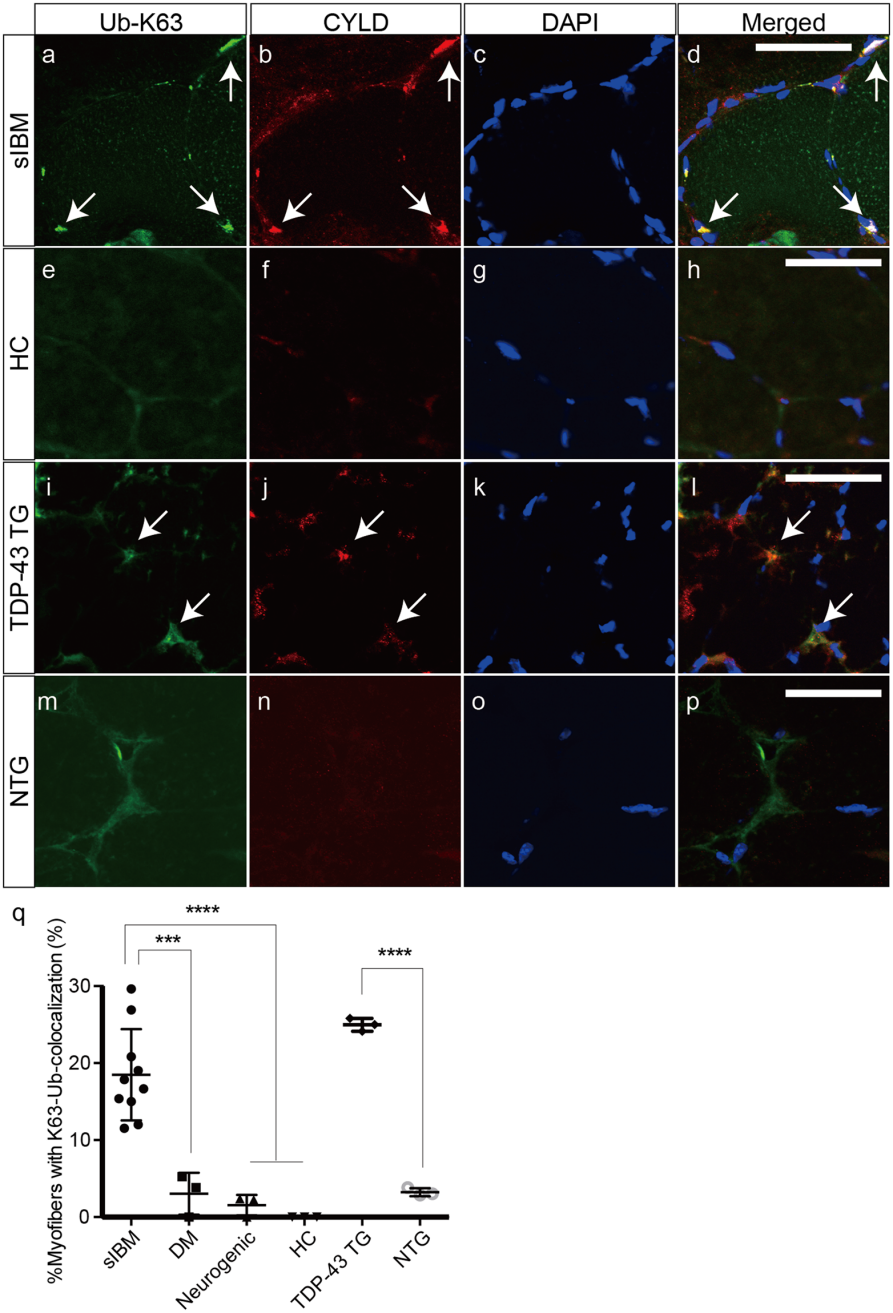
Colocalization of CYLD with Lys63-linked ubiquitin in muscle fibres of sIBM patients and TDP-43 TG mice. (**a**–**p**) Confocal microscopic analysis of the localization of K63-linkage-specific polyubiquitin (**a**,**e**,**i**,**m**) and CYLD (**b**,**f**,**j**,**n**) in muscles of patients with sIBM (**a**–**d**), healthy control (**e**–**h**), and TDP-43 TG (**i–l**) or NTG mice (**m–p**). Nuclei were stained with DAPI. Merged images are presented in (**d**,**h**,**l**). Scale bars = 50 μm. (**q**) Percentages of myofibres with colocalization of K63- polyubiquitin and CYLD in each group. n = 3–10 per group. ***P < 0.001; ****P < 0.0001.

Because Lys63-linked ubiquitin is involved in the selective autophagy of sIBM, we then investigated whether Lys63-linked ubiquitin cooperated with CYLD in the nuclear and perinuclear areas of sIBM and TDP-43 TG mice. Confocal microscopic analysis demonstrated frequent colocalization of Lys63-linked ubiquitin and CYLD in the nuclear and perinuclear areas of muscle fibres from sIBM-affected tissues (Fig. 5a–d) and TDP-43 TG mice (Fig. 5i–l). In contrast, less colocalization was observed in the control and muscle samples from NTG mice (Fig. 5e–h,m–p). The percentage of myofibres with colocalization of Lys63-linked ubiquitin and CYLD was considerably higher in the muscle samples of patients with sIBM and TDP-43 TG mice, compared to the other experimental groups (Fig. 5q). This indicates that CYLD might be involved in selective autophagy in the nuclear and perinuclear regions of muscle fibres of sIBM tissues, and might be associated with p-TDP-43, p-p62, and Lys63-linked ubiquitin.

### Comprehensive proteomic analysis using myofibres with rimmed vacuoles of sIBM patients

**Figure 6:**
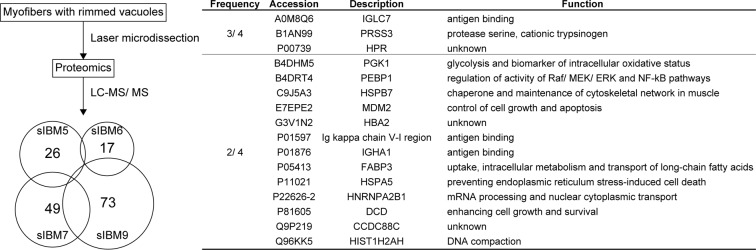
Comprehensive proteomic analysis of myofibres with rimmed vacuoles from sIBM patients. Myofibres with rimmed vacuoles from sIBM patients 5, 6, 7, and 9 were analysed using LC-MS/MS. The analysis identified 26, 17, 49, and 73 peptides in the sarcoplasm with rimmed vacuoles of sIBM patients 5, 6, 7, and 9, respectively. Among these peptides, the peptides detected in more than 2 patients were listed.

Then, we conducted proteomic analysis to identify the components of degenerating myofibres from sIBM patients. We dissected the myofibres with rimmed vacuoles using a laser microdissector, digested the samples with trypsin, and analysed them with LC-MS/MS. The analysis identified expression of 26, 17, 49, and 73 peptides in the sarcoplasm with rimmed vacuoles of sIBM patients 5, 6, 7, and 9, respectively (Fig. 6). Among these peptides, IGLC7, PRSS3, and HPR were detected in 3 of 4 sIBM patients; whereas IGLC7, PRSS3, HPR, PGK1, PEBP1, HSPB7, MDM2, HBA2, Ig kappa chain V-I region, IGHA1, FABP3, HSPA5, HNRNPA2B1, DCD, CCDC88C, and HIST1H2AH were observed in 2 of 4 patients. The peptides were generally classified into families of immunoglobulin; cell growth and survival, or apoptosis; ER-resident chaperone for misfolded proteins; and DNA/RNA regulation. Of note, PEBP1, also known as Raf-1 kinase inhibitor protein, has been identified as a negative regulator of the NF-κB/SNAIL circuit^19,20^, although we failed to detect CYLD expression in these samples.

### Downregulation of CYLD and reduced cell viability in cell models treated with ER stress inducers

**Figure 7:**
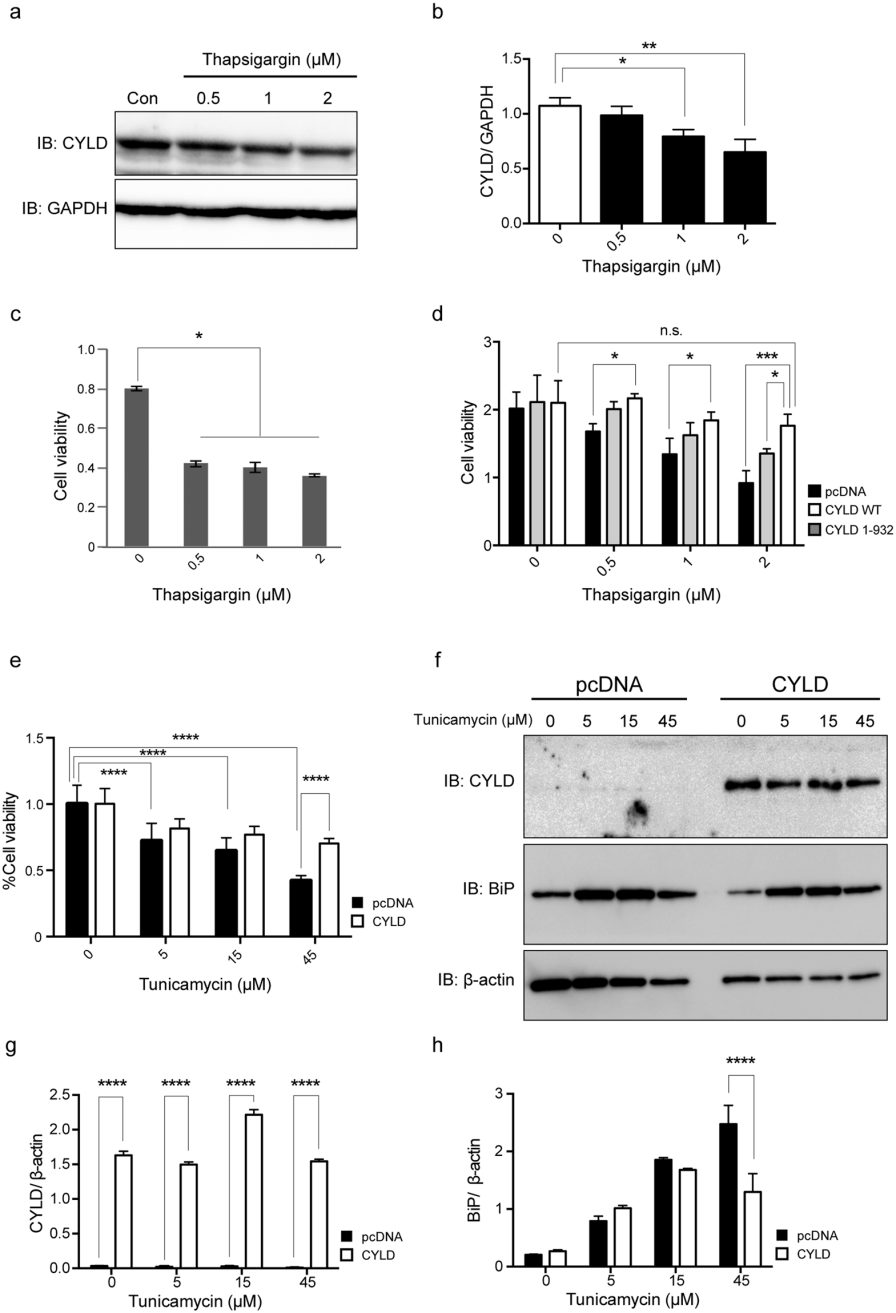
Downregulation of CYLD and reduced cell viability in cell models treated with ER stress inducers. (**a**) Representative immunoblots of CYLD and glyceraldehyde-3-phosphate dehydrogenase (GAPDH) in C2C12 cell lysates 24 hours after treatment with thapsigargin (0, 0.5, 1, and 2 µM). (**b**) Quantification of CYLD band intensity normalized to GAPDH. *P < 0.05; **P < 0.01. (**c**) Tetrazolium-based colorimetric (MTS) assay in C2C12 cells at 24 h after treatment with thapsigargin (0, 0.5, 1, and 2 µM). n = 3 per group. *P < 0.05. (**d**) MTS assay in C2C12 cell lysates at 48 h after transfection of wild-type CYLD (CYLD WT), deubiquitinase-deficient mutant of CYLD (CYLD 1–932), or pcDNA3 and 24 h after treatment with thapsigargin (0, 0.5, 1, and 2 µM). n = 3 per group. *P < 0.05; ***P < 0.001; n.s. not significant. (**e**) MultiTox-fluor multiplex cytotoxicity assay in RD cells with or without CYLD overexpression at 24 h after treatment with tunicamycin (0, 5, 15, and 45 µM). n = 3 per group. ****P < 0.0001. (**f**) Representative immunoblots for CYLD, BiP, and β-actin using RD cell lysates with or without CYLD overexpression after treatment with tunicamycin (0, 5, 15, and 45 µM). n = 3 per group. (**g**) Quantification of CYLD band intensity normalized to β-actin. ****P < 0.0001. (**h**) Quantification of BiP band intensity normalized to β-actin. ****P < 0.0001.

Because CYLD negatively regulates activation of NF-κB signalling pathways by regulating the IκB kinase pathway^16^, which might be pivotal molecules for the pathogenesis of sIBM, we investigated whether overexpression of CYLD can affect muscle toxicity in the cell models treated by ER stress inducers. Induced ER stress activates the signalling for muscle fibre atrophy through activation of NF-κB in differentiated cultured muscle fibres^14^. We first treated mouse myoblast cells (C2C12) with thapsigargin (0–2 µM). Of note, expression levels of CYLD decreased (Fig. 7a,b and Supplementary Information) and cell viability was reduced in cells treated with thapsigargin (Fig. 7c). We next investigated the effect of CYLD on cell toxicity induced by thapsigargin. Control cells transfected with pcDNA3, wild-type CYLD (CYLD WT), and mutant CYLD lacking catalytic domain (CYLD 1–932) had similar cell viabilities (Fig. 7d). However, CYLD WT overexpression substantially improved cell viability following treatment with thapsigargin (0.5–2 µM) (Fig. 7d). CYLD 1–932 has been shown to severely reduce deubiquitinase activity^16^. When CYLD 1–932 was overexpressed, the effect on cell toxicity induced by thapsigargin was diminished, suggesting that protective effect of CYLD might be dependent on deubiquitinase activity. We also used rhabdomyosarcoma cells treated with tunicamycin to confirm the above results. Cell viability considerably decreased after treatment of tunicamycin (5–45 µM) (Fig. 7e) but CYLD overexpression significantly improved cell viability when cells were treated with 45 µM tunicamycin (Fig. 7e). Overexpression of CYLD also reduced the expression level of BiP, a marker for ER stress, following treatment with 45 µM tunicamycin compared to control cells (Fig. 7f–h, and Supplementary Information).

## Discussion

We have demonstrated that CYLD localized in the nuclear and perinuclear regions of degenerative myofibres with rimmed vacuoles in sIBM-affected tissues. CYLD showed localization associated with p-TDP-43, p-p62, and Lys63-linked ubiquitin in the myofibres of sIBM patients and wild-type TDP-43 mice. Although ER stress inducers reduced the expression levels of CYLD and decreased cell viability, wild-type CYLD substantially improved cell viability, possibly by reducing the misfolded protein load.

CYLD localizes primarily in the cytoplasm and perinuclear region of various cell types^21^. In cardiovascular research, CYLD has been shown to interact with p62 directly and CYLD can directly inactivate HDAC6, thereby controlling autophagy^22^. Thus, it has been suggested that CYLD can regulate the autophagic pathway that clears damaged protein aggregates and reduces the chance of lesion formation by its ability to control the cell cycle, inflammation, and autophagic factors in vascular diseases^21^. Consistent with these findings, our observation that CYLD overexpression reduced expression levels of BiP, an ER stress marker, suggests that CYLD promotes autophagic clearance of misfolded proteins resulting from ER stress inducers in cell models. Because insoluble protein cannot be detected by our proteomic methods, CYLD might be aggregated and insolubilized with TDP-43, phosphorylated-TDP-43, phosphor-p62 and Lys63-linked ubiquitin, which could explain why we failed to detect CYLD expression in our proteomic samples from degenerating myofibres of sIBM patients.

There is some contention as to whether sIBM is primarily an inflammatory or a degenerative myopathy^23^. The inflammatory changes, specifically those that mimic polymyositis, are more often observed at the early stages of the disease, whereas the degenerative changes are seen more commonly in the advanced stages^24^. Delineating the relationship between inflammatory and degenerative changes in sIBM is a prime concern in the field of clinical myology. Recently, two independent groups identified the target antigen of sIBM autoantibodies as cytosolic 5ʹ-nucleotidase 1 A (cN1A) in the plasma and serum samples of patients with sIBM^25,26^. Although the pathogenic role of the anti-cN1A autoantibodies has not been precisely determined, our previous experiments revealed p62-positive sarcoplasmic aggregates in the myofibres of mice injected with anti-cN1A-positive sIBM IgG in an *in vivo* passive immunization model^27^. Thus, both inflammation and degeneration may provoke a vicious circle, reinforcing the myopathic process in sIBM. Although we have not shown direct evidence for this, reduced expression of CYLD may fail to negatively regulate NF-κB signalling pathways, thus promoting an inflammatory mechanism in sIBM pathogenesis.

Necroptosis is a novel form of programmed cell death that is characterized by necrosis-like morphological changes, and is triggered by RIP kinases RIPK-1 and RIPK3, and pseudokinase mixed lineage kinase domain-like (MLKL)^28^. Necroptosis is involved in various pathological conditions, such as ischemia-reperfusion injury in the heart and brain, and injury-induced inflammatory diseases and neurodegenerative diseases^28^. Recently, CYLD has been identified as an essential factor for tumour necrosis factor-induced necroptosis^29,30^. Although it remains unclear whether necroptosis is involved in the pathogenesis of sIBM, the possibility that reduced expression of CYLD prevents necroptosis should be investigated.

The present study has a few limitations. First, the wild-type TDP-43 transgenic mouse model bears little resemblance to sIBM, such as TDP-43-positive inclusions; however, the model lacked inflammatory cell infiltrates as well as the presence of rimmed vacuoles^31^. Second, CYLD may be another unfortunate bystander in the underlying pathology of IBM, because many proteins are accumulated in the myofibres of sIBM^6^ tissues, and as a result it is somewhat difficult to identify which are primary or secondary events.

In conclusion, dysregulation of CYLD may reinforce myodegeneration in the pathophysiology of sIBM by attenuating autophagic clearance. Further investigation is required for a better understanding of the role of CYLD in the pathogenesis of sIBM. Regulating CYLD in myofibres might be a novel therapeutic strategy in treating sIBM.

## Methods

### Patients and muscle biopsies

We investigated muscle biopsy samples from 11 patients with sIBM, 3 with polymyositis, 3 with dermatomyositis, 3 with neurogenic atrophy, and 3 healthy controls (Table 1). Muscle biopsy was conducted for diagnostic purposes after obtaining informed consent from all the patients. Diagnoses of sIBM were based on the ENMC2011 criteria as previously described^32^. All muscle samples were first examined by routine histological techniques for diagnostic purposes. Fresh frozen tissues were kept at −80 °C. The study was approved by the Ethics Committee of the Kumamoto University Hospital, Japan. All methods were carried out in accordance with the relevant guidelines and regulations.

### Generation of TDP-43 TG mice

TDP-43 TG mice that express wild-type human TDP-43 by a creatine kinase 8 promoter^31^ were used for these experiments. TDP-43 TG mice showed myopathic changes associated with elevation of serum myogenic enzymes at 18 months of age: fibre size variation, tubular aggregates, TDP-43 aggregates, and upregulation of ER stress pathway. TDP-43 TG and NTG mice at 18-month old were sacrificed, and the thigh muscles were removed and fresh-frozen at −80 °C. All animal research was approved by the Kumamoto University Committee on Animal Research, and was performed in accordance with the relevant guidelines and regulations required by the Kumamoto University, Japan.

### Cell culture and transfection

C2C12 cells (mouse myoblasts) or RD cells (rhabdomyosarcoma) were purchased from Riken Bioresource Center (Tsukuba, Japan). The pcDNA3-FLAG-CYLD vector was provided by Dr. Satoru Shinriki and Dr. Hirofumi Jono (Kumamoto University Hospital, Kumamoto, Japan). The fragment of catalytic mutant CYLD (CYLD 1–932) was generated by PCR, and subcloned into the pcDNA3 vector. Cells were transfected with plasmids using Lipofectamine 2000 (Life Technologies, Carlsbad, USA). Twenty-four hours after transfection, cells were shifted to fresh medium supplemented with foetal calf serum (10% vol/vol) containing thapsigargin (0.5, 1, and 2 μM; Sigma-Aldrich, St. Louis, USA) or 5, 15, and 45 μM of tunicamycin (Sigma-Aldrich) for 24 h.

### Immunohistochemical analyses

The muscle specimens were fixed with paraformaldehyde (4%), and blocked with normal donkey serum (5%)/Triton-X in phosphate-buffered saline (0.1%). The primary antibodies were as follows: rabbit anti-CYLD (1:250; Sigma-Aldrich, Cat. No. SAB4200060); rabbit anti-CYLD (H-419) (1:200; Santa Cruz Biotechnology, Santa Cruz, USA, Cat. No. sc-25779); mouse anti-CD4 (prediluted; Nichirei Biosciences, Tokyo, Japan, Cat. No. 413181); mouse anti-CD8 (prediluted; Nichirei Biosciences; Cat. No. 413201); rabbit anti-TDP-43 (1:500; ProteinTech, Chicago, USA, Cat. No. 10782-2-AP); rabbit anti-OPTN (1:250; Cayman Chemical, Ann Arbor, USA, Cat. No. 100000); mouse anti-phosphorylated TDP-43 (p-TDP-43; pS409/410) (1:3000; Cosmo Bio, Tokyo, Japan, Cat. No. CAC-TIP-PTD-M01); mouse p-p62/SQSTM1 (Ser351) (1:500; Medical & Biological Laboratories, Nagoya, Japan, Cat. No. M217-3); and mouse anti-polyubiquitin (Lys63-linkage-specific) (1:100; Enzo Life Sciences, Farmingdale, USA, Cat. No. BML-PW0600). Immunolabeled proteins were visualized by the avidin-biotin-peroxidase complex method with a Vectastain Elite ABC Kit (Vector Laboratories, Burlingame, USA), or anti-mouse Ig antibody-conjugated Alexa Fluor 488 and anti-rabbit Ig antibody-conjugated Alexa Fluor 594 (1:200 dilution; Life Technologies, Cat. No. A21202 and A21207). Nuclei were stained with 4’,6-diamidino-2-phenylindole (DAPI; Vector Laboratories), and observed with confocal microscopy (FV1200, Olympus, Tokyo, Japan).

### Proteomic analysis with laser-microdissection

The procedure for proteomic analysis was described previously^31^. In brief, muscle biopsy specimens from sIBM patients (no. 5, 6, 7, and 9) were dried overnight. Myofibres with rimmed vacuoles (1,000,000 µm^2^) were collected using the LMD7000 system (Leica Microsystem, Wetzlar, Germany). Collected tissue samples in Tris (10 mM), EDTA (1 mM), and Zwittergent (0.002%) were heated at 98 °C for 90 min, sonicated for 90 min, and digested with 1.5 μl trypsin (1 mg/ml; Promega, Madison, USA) overnight at 37 °C. Digested peptides were analysed using nanoflow reversed-phase LC–MS/MS (LTQ Velos Pro; Thermo Fisher Scientific, Waltham, USA).

### Protein preparation and western blot analysis

Cells were solubilized with radioimmunoprecipitation assay (RIPA) buffer (Tris-HCl (50 mM), pH 8.0; NaCl (150 mM); NP-40 (1%); sodium deoxycholate (0.5%); and SDS (0.1%)) and protease inhibitor cocktail (Promega, Madison, WI, USA). The primary antibodies were as follows: rabbit anti-CYLD (1:250; Sigma-Aldrich, Cat. No. SAB4200060), mouse anti-GADD153 (B-3) (1:200; Santa Cruz Biotechnology, Cat. No. sc-7351), anti-glyceraldehyde-3-phosphate dehydrogenase (GAPDH) (1:1000; Medical & Biological Laboratories), mouse anti-BiP/GRP78 (1:250; BD Biosciences, San Jose, USA), and mouse anti-β-actin antibody (1: 10,000; Sigma-Aldrich). After incubation with the primary antibodies for 16 h at 4 °C, the membranes were reacted with the corresponding horseradish peroxidase (HRP)-conjugated secondary antibodies (GE Healthcare, Buckinghamshire, UK) for 1 h. Bands were detected by ECL prime western blotting detection system (GE Healthcare) with ImageQuant LAS-4000 mini EPUV (Fuji film, Tokyo, Japan). Grouping of blots were cropped from different parts of the same gel, and intensity of bands was quantified by ImageJ 1.50i software (National Institutes of Health, NIH).

### Measurement of cell viability

Cells transfected with CYLD or pcDNA3 vectors were analysed using a tetrazolium-based colorimetric assay (3-[4,5-dimethylthiazol-2-yl]- 5-[3-carboxymethoxyphenyl]-2-[4-sulfophenyl]-2H-tetrazolium [MTS] test) (CellTiter 96 AQueous one solution cell proliferation assay kit; Promega) or a MultiTox-fluor multiplex cytotoxicity assay kit (Promega) at 48 h after transfection.

### Statistics

All values are expressed as the mean ± SEM. Differences among means were analysed using one-way or two-way ANOVA. When ANOVA analysis revealed significant differences, pair-wise comparisons were performed using Tukey post-hoc test.

## Supplementary information

Supplementary information

## Data Availability

All data generated or analysed during the current study are included in this published article.
