# Supplementary material for: CYLD dysregulation in pathogenesis of sporadic inclusion body myositis
Source: Sci Rep. 2019 Aug 12;9:11606. doi: 10.1038/s41598-019-48115-2 (PMC6690995; doi:10.1038/s41598-019-48115-2)
Supplement: Supplementary file 1 — Supplementary information [file 41598_2019_48115_MOESM1_ESM.docx]

**CYLD dysregulation in pathogenesis of sporadic inclusion body myositis**

Satoshi Yamashita*, Yoshimasa Matsuo, Nozomu Tawara, Kentaro Hara, Masanori Yamamoto, Tomo Nishikami, Kensuke Kawakami, Xiao Zhang, Ziwei Zhang, Tsukasa Doki, Yukio Ando

**Supplementary figure.**

**
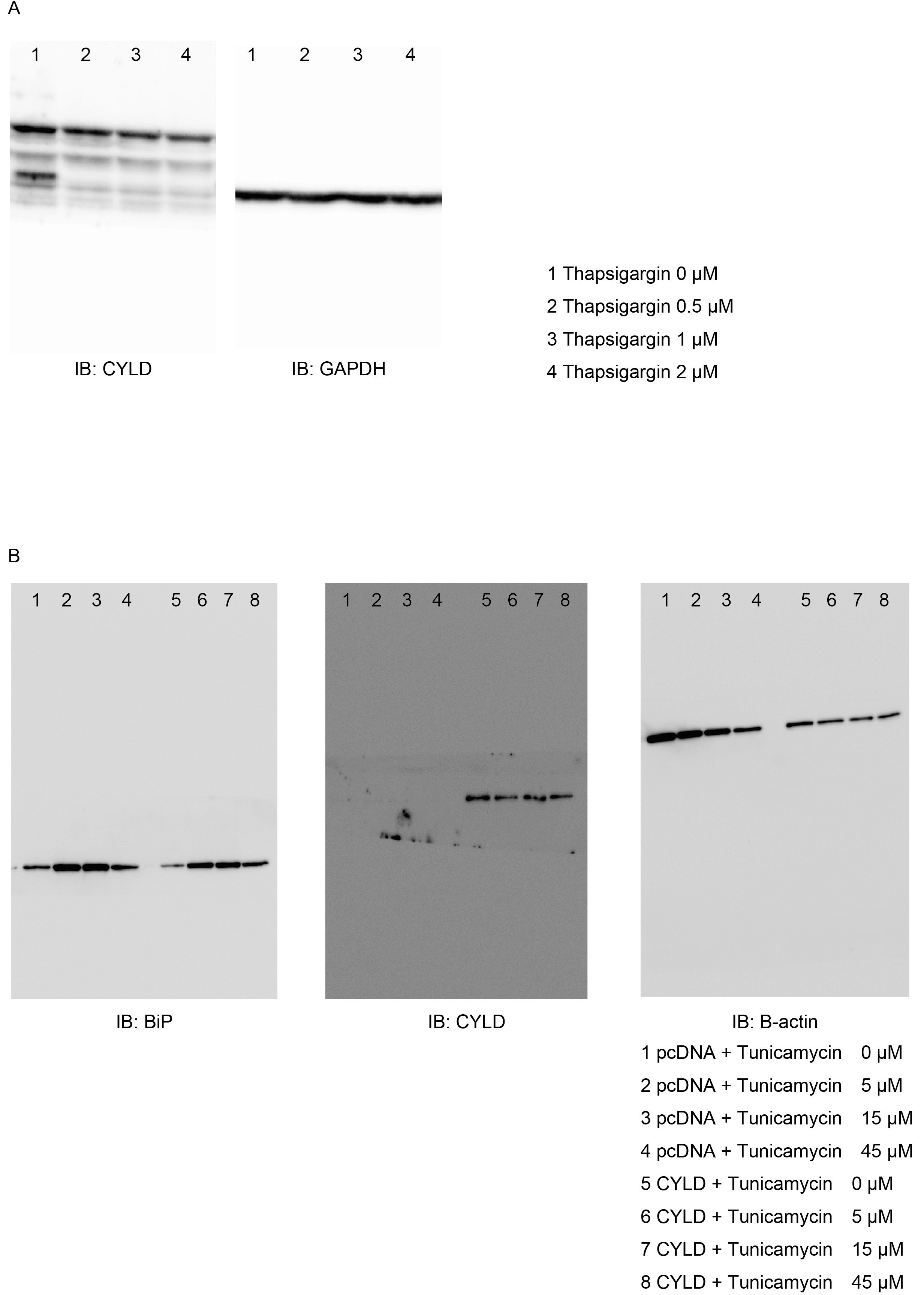
**

(A) Full images of representative immunoblots of CYLD and glyceraldehyde-3-phosphate dehydrogenase (GAPDH) in C2C12 cell lysates 24 hours after treatment with thapsigargin at concentrations of 0 (lane 1), 0.5 (lane 2), 1 (lane 3), and 2 µM (lane 4). (B) Full images of representative immunoblots for CYLD, BiP, and β-actin using RD cell lysates with or without CYLD overexpression after treatment with tunicamycin at concentrations of 0 (con), 5, 15, and 45 µM. Lane 1: pcDNA + tunicamycin 0 µM; lane 2: pcDNA + tunicamycin 5 µM: lane 3: pcDNA + tunicamycin 15 µM; lane 4: pcDNA + tunicamycin 45 µM: lane 5: CYLD + tunicamycin 0 µM; lane 6: CYLD + tunicamycin 5 µM; lane 7: CYLD + tunicamycin 15 µM; lane 8: CYLD + tunicamycin 45 µM.
